# Healthcare telemonitoring and business dynamics: challenges and opportunities for SUS

**DOI:** 10.11606/s1518-8787.2020054001996

**Published:** 2020-06-19

**Authors:** Antonio da Cruz Paula, José Manuel Santos de Varge Maldonado, Carlos Augusto Grabois Gadelha

**Affiliations:** I Cnpq Brasil Grupo de pesquisa cadastrado no Cnpq: apoio à gestão de tecnologia, inovações e produtos estratégicos para o Sistema de Saúde.Brasil; II Fundação Oswaldo Cruz Escola Nacional de Saúde Pública Sérgio Arouca Rio de JaneiroRJ Brasil Fundação Oswaldo Cruz. Escola Nacional de Saúde Pública Sérgio Arouca. Rio de Janeiro, RJ, Brasil

**Keywords:** Noncommunicable Diseases, prevention & control, Telemonitoring, Health Care Sector

## Abstract

**OBJECTIVE:**

To point out challenges and opportunities for the Brazilian Unified Health System (SUS) with the use of telemonitoring to face the increasing costs of non-communicable chronic diseases, based on its general panorama in Brazil, business dynamics and reapplication of data from American studies.

**METHODS:**

Quali-quantitative approach with exploratory research. The field work focused on the analysis of the national market from private companies, since no experiences or studies related to this theme were identified in the SUS. To analyze the panorama and market dynamics, we investigated the offer of this technology based on the products and services available and their demand by reference hospitals the ten largest private health plan companies. To support the central discussion, we analyzed the reduction of costs with hospital admissions by the SUS due to chronic non-communicable diseases sensitive to telemonitoring (HCDST), using data from Datasus and some American studies from the MEDLINE/PubMed database.

**RESULTS:**

Although in the embryonic phase, business agents search for new business opportunities, whereas public initiatives for the use of telemonitoring in collective health seem inexistent. The reapplication of U.S. data would reduce spending on HCDST and provide benefits, such as the reduction in emergency room care, acute hospitalizations, readmissions and home care time, among others, which point to even greater economic gains.

**CONCLUSIONS:**

The development of a major project to reduce HCDST using this technology has the potential to advance in a comprehensive network of primary care, contribute to a greater dynamism of the national productive and innovative base and induce innovations along the chain of this emerging industry.

## INTRODUCTION

Telemonitoring is one of the most important applications of telemedicine, characterized by telemonitoring of health data of a patient by a specialized center for monitoring, interpretation and analysis. Dehospitalization, preventive health, early diagnosis, increased life expectancy, better comfort and increased worked hours are among the benefits of this technology. It has been considered important to address the growth in the costs of chronic non-communicable diseases (CNCD) by the national health systems, one of the main problems in the provision of health services worldwide^1^.

CNCD are a global concern, impacting the goals established in the Millennium Development Goals, as they are responsible for the main causes of death in the world, with repercussions on the loss of quality of life and limitation of work and leisure, as well as on the economic impacts for families and health systems. Therefore, the United Nations (UN) has instructed countries to develop action plans for their prevention and control^4,5^.

Primary health care plays an important role in their prevention and control, since some CNCD are preventable by conditions sensitive to primary care^6,7^. Therefore, countries seek different approaches, among which telemonitoring has stood out as an important alternative.

Brazil faces significant challenges in the construction of the Unified Health System (SUS) due to the country’s continental dimensions and population of more than 200 million people, which hinders the compliance with the constitutional precept of health universalization. Moreover, the complex demographic and epidemiological transitions imply greater efforts that impact the health budget. The increase in CNCD accounts for 75% of SUS expenses and 72% of deaths, of which 31.3% are due to diseases of the circulatory system, 16.3% of cancer, 5.2% of diabetes and 5.8% of respiratory diseases, being the four most representative groups^4,8^.

Despite the experiences and important studies from the Telehealth Network Program (Telehealth), the scientific production in the specific field of telemonitoring that seeks to demonstrate the benefits arising from its use is insufficient, which includes the analysis of costs, given its undeniable importance for public and private health systems^7,9^. One example is a recent study conducted in Brazil with older adults in chronic condition, which pointed out a positive relationship between the use of telemonitoring and adherence to drug treatment^12^.

However, we observed a development and offer of solutions for the Brazilian market, which shows the interest and expectation of business agents in the growth of demand, whereas public policies, in turn, are scarce in this area. This fact points to the need for greater participation of the State, given the risk of the opportunities opened by telemonitoring being appropriated exclusively by business strategies.

Thus, the objective of this article is to identify the challenges and opportunities for the SUS due to the use of telemonitoring, based on business and institutional dynamics and economic benefits.

## METHODS

Our study fits into the qualitative-quantitative approach with exploratory research. A literature review allowed a qualitative analysis with developments in the quantitative approach to estimate the cost reduction with the use of telemonitoring by the SUS^13^.

Within the Scope of the SUS, no experiences or studies related to this theme were identified in the search in the SciELO, Capes and Google Scholar databases, as well as in the analysis of projects between 2009 and 2020 of the SUS Institutional Development Support Program (Proadi)^14^. Therefore, the fieldwork focused on the analysis of the national market from private companies.

Regarding the offer, a survey was conducted to identify companies that sell specialized services and telemonitoring products in the Brazilian market, based on searches in the Google search with the descriptors “remote health monitoring,” “health management,” “homecare” and “health teleassistance,” while suppliers of products were identified among the exhibiting companies in the largest fair of hospital equipment in Brazil in 2017^15^.

Regarding the demand, we opted for the reference hospitals recognized by the Brazilian Ministry of Health (MS) in the triennium of 2018–2020, i.e., the Health Entities of Recognized Excellence (HERE), and the ten largest private health plan companies were selected, which establish a competitive *modus operandi*^16,17^.

To support the central discussion, we analyzed the reduction of costs with hospital admissions by the SUS due to CNCD most impacted by telemonitoring (cardiovascular and respiratory diseases, and diabetes), hereinafter “hospitalizations for chronic diseases sensitive to telemonitoring (HCDST),” a subgroup of hospitalizations due to CNCD (HCNCD), a category already existing and monitored by the MS^1,4,18^. Datasus and some studies from the MEDLINE/PubMed database, identified by the descriptors “telemedicine,” “telehealth” and “ehealth” associated with the terms “economic benefit monitoring” and “remote patient monitoring” were also used. Finally, the calculations of cost reduction with HCDST were obtained from the replicability of the most conservative data from these studies.

## RESULTS

We present a framework of telemonitoring in Brazil with data from the main policy initiatives, regulatory issues and the national market, as well as some estimates of economic benefits for the SUS with the use of telemonitoring.

### Telemonitoring Framework in Brazil

There are few actions to expand its use in Brazil, especially by the SUS, despite the immense benefits pointed out by specialists and the evolution of telemedicine in the country in recent years, resulting from incentives of research promotion agencies and important government actions that resulted in the formation of infrastructure, teams and research centers in several academic institutions in the country. Regarding public policies, it can be said that in Brazil there were some initiatives that culminated essentially in the projects University Telemedicine Network (Rute) and Telehealth, both with a focus on telemedicine to support and improve the quality of Primary Health Care^9,10^.

Currently, Telehealth is implemented in all states of the country and is an important instrument of innovation for the Family Health Program (FHP). Despite the emphasis given in some states, such as the remote electrocardiogram service developed in the state of Minas Gerais, teleradiography (TeleRX) in Rio de Janeiro, telediagnosis for chronic respiratory diseases and obstetric ultrasounds in Rio Grande do Sul and teleducation in São Paulo, among others, national actions in the field of telemonitoring are not identified.

However, although the Strategic Action Plan for Coping with CNCD in the MS provides for the use of telemonitoring, and studies point to its benefits, there are no initiatives in the field of public health policies in Brazil, as well as formal studies of the Ministry of Health for an analysis of its cost-benefit, although several other countries, such as members of the European Union (EU), have this application as one of the main focuses in the strategy to combat the problems arising from this group of diseases^4,19^.

Regarding regulatory aspects, there is a wide variety in the positioning of federal councils regarding the use of telemedicine by professionals and service providers. Some councils have a liberal position, but no regulations for the provision of distance services, such as those of physical education and social services; there are others with significant flexibility, such as those of psychology and nursing; and some others are still very reactive, such as the councils of physiotherapy, nutrition and medicine^20^. Unlike other applications of telemedicine, Telemonitoring has no explicit regulatory restriction in different health specializations.

The case of the Federal Council of Medicine (FCM) deserves a more detailed analysis. With resolution no. 2,227 of 2018 published in February 2019, the FCM expanded the attributions of telemedicine, enabling distance medical services vis-à-vis, that is, teleconsultation. However, the FCM opted to postpone its entry into force due to the numerous criticisms from physicians, representative entities and regional councils, especially regarding the insufficient participation in the drafting of this resolution, the early release by some private companies of new services based on teleconsultation, prohibited until then by resolution No. 1,643 of 2002.

Although this discussion has as its central element the teleconsultation, it is reflected in telemonitoring regarding private initiative, for its responsiveness, based on the perspective of occupation of this market with the offer of integrated service packages. This point was illustrated the anticipation of announcement of teleconsultation by the hospital Albert Einstein in early February 2019. In the field of public health, in turn, regulatory decisions that do not involve all stakeholders with extensive discussions and reflections on their impacts may distort comprehensive care projects and further weaken the SUS.

Specialized services and products are offered for the national market, as well as a wide variety of devices, which indicates a potential for the commercialization of this technology in the country. Chart 1 shows companies in Brazil that offer specialized services and products within the scope of telemonitoring, the federative unit in which they are located, their application and customers^4,11^.

**Chart 1:** Companies providing specialized services and telemonitoring products: field survey.


Websites	Specialized service providers				

	Company	State	Product/services	Application	Customers

http://www.ocuidadorvirtual.com.br/	24/7 Care	SP	Teleassistance in general, medical guidance at a distance and ambulance call	Electronic bracelet with alarm button for care of older adults	Retail market
https://www.athoscare.com.br/	Athoscare	SP	Home care service with basic telemonitoring (by phone)	Patients with diabetes, hypertension and Alzheimer’s, either bedridden or with some difficulties	Retail market
http://lincare.com.br/	Lincare	MG	Remote cell phone monitoring for older adults	Electronic wristband for measuring sleep, heart rate, blood pressure and steps/distance covered	Retail market
https://qualirede.com.br/	Qualirede	SC	Specialized in health insurance management	Artificial intelligence, data analytics, data science and predictive analysis – health management of 65,000 chronic patients	Corporate market
http://www.telehelp.com.br/	Tele Help	SP	Teleassistance in general, medical guidance at a distance and ambulance call	Electronic bracelet with alarm button for care of older adults	Retail market
http://unicaresaude.com.br/	Unicare Saúde	SP	Service provider in the segment of high complexity home care	Uses the i9Access solution	Health plan operators and retail market
	
	**Product suppliers**				

	**Company**	**State**	**Service**	**Application**	**Focus**
	
	Apple	Global	*Saúde* app and Apple Watch	Preventive health with a focus on encouraging and controlling food, physical activities, sleep and relaxation activities	Retail market – users of smartphones
	Bioaps	SP	PRM – Patient Relantionship Management	User empowerment and prevention	61,000 patients at the health insurance company São Francisco
	Cisco/Nexa	Global	SmartCare	Concept of collaboration platform with a call center that encourages the adoption of healthy habits, compliance with prescriptions and monitors the health conditions of customers	Hospitals, clinics and health professionals
	Fitbit	Global	Fitbit Smart Bracelets	Physical activity monitoring bracelets	Retail market
	i9Access	RS	Telemonitoring management system	Telemonitoring and empowerment	*Unicare Saúde* – home care service provider
	Lifemed	SP	Central Lifeview capable of monitoring vital signs for up to 32 patients	Tele-homecare, preventive health and CNCD	Hospitals, clinics and health professionals
	Mcare	RJ	Mobile system-based telemonitoring system	Obtains and manages device data via Bluetooth, passing on to healthcare professionals by email, voice, or text	Hospitals, clinics and health professionals
	Philips	Global	IntelliVne patient monitoring center	Remote access to patient information for support in critical decisions	Hospitals, clinics and health professionals
	Signove	PB	Sig Health platform, a telemonitoring system integrated with various devices	Tele-homecare, preventive health and CNCD	Hospitals, clinics and health professionals
	Samsung	Global	S Health app	Preventive health with a focus on encouraging and controlling food, physical activities, sleep and relaxation activities	Retail market – Samsung smartphone users
	Ventrix	SP	Telemedicine systems	Telemonitoring of babies and tele-ECG	Retail market and healthcare professionals
	Xiaomi	Global	Smart watches	Smart watches for monitoring physical activity	Retail market

Source: Own elaboration based on Hospitalar^15^, Maldonado, Marques and Cruz^11^ and institutional websites of the companies.

There is diversity regarding the modality of providing specialized services, such as homecare, health management and telemonitoring service providers for older adults. As for the technologies used, the use of telephone, *wearables* or smart clothing such as electronic bracelets, as well as the application of artificial intelligence and big data for predictive analysis and health management.

Regarding the products, we observed, in the market, an offer of solutions aligned with the main strategies pointed out by the literature for their use by patients with CNCD: education or self-care, telecare and management or monitoring. However, the main consumer market is retail, which indicates that service providers have not yet adopted telemonitoring as a tool. On the other hand, it is a sign that their acceptance by the end user grows. These first movements of the private sector signal prospects for market growth, in which new business opportunities, service expansion and increased health costs due to CNCD, among other factors, help explain the growing interest in this emerging industry. Despite the dissemination of studies in other countries that point to positive impacts of its use regarding the improvement in quality of life and cost reduction in the provision of health services to patients with CNCD, some initiatives of private companies in this area focus mainly on telemonitoring without the use of innovative technologies that would allow remote telemonitoring in real time.

Chart 2 shows the most important private health service providers in the country, which have developed initiatives for health prevention and management in their businesses, with the perspective of improving the quality of life and self-care of patients, especially of chronic patients, albeit with different objectives. Hospitals bet on the development of a new market, preventive medicine, for which they built structures with multidisciplinary teams and equipment, having the commercialization of check-up exams as a central element. On the other hand, health insurers have similar structures for health prevention and management, aiming mainly at reducing costs.

**Chart 2:** Main initiatives of private service providers.


Reference hospitals				

Company	State	Service	Application	Focus

Albert Einstein^a^	SP	Tele babycare, *telecessação do tabagismo, Einstein em movimento and bem estar Einstein*	Babies, smokers, guided physical activities, positive psychology	Self-care as a result of the sale of check-up and preventive examinations
Albert Einstein^a^	SP	Teleconsulta	Companies, patients and other health care providers	Creation of new integrated service packages and market expansion
Sírio Libanês	SP	Health monitoring and check up	Preventive medicine and chronic patients	Self-care as a result of the sale of check-up and preventive examinations
Hospital do Coração	SP	Check-up program for teleconsultation and telediagnosis for SUS’s patients	Preventive medicine and chronic patients	Self-care as a result of the sale of check-up and preventive examinations to customers – new business with telemedicine services for SUS and other hospitals
Hospital Alemão Oswaldo Cruz	SP	Care model	Preventive medicine	Self-care as a result of the sale of check-up and preventive examinations

**Insurers and operators of private health plans**				

**Company**	**State**	**Service**	**Application**	**Focus**

Amil	National	High-risk patient management and family health strategy	Chronic patient monitoring, single gateway for patients with FHS and incentive to self-care	Improving quality of life and reducing costs with beneficiaries
Bradesco Saúde	National	*Juntos pela saúde*	Monitoring of chronic and anti-smoking patients	Improving quality of life and reducing costs with beneficiaries
Cassi	National	*Estratégia da família*	Health promotion and disease prevention	Rational use of the network of accredited and specialized services
Hapvida	Northeast	HapPrev and *Viva Leve* Programs	Preventive medicine, self-care and continuous monitoring of HapPrev participants – chronic education and monitoring with *Viva Leve*.	Improving quality of life and reducing costs with beneficiaries
Intermedica	National	Support for patients with chronic diseases	Proactive monitoring and self-care	Improving quality of life and reducing costs with beneficiaries
Nacional Unimed	National	Comprehensive health care strategy and health management program	Self-care and self-care engagement monitoring for chronic patients	Improving quality of life and reducing costs with beneficiaries
Sulamérica	National	*Saúde ativa* program and sharecare app	Innovative technologies to monitor health conditions and engage beneficiaries in self-care activities	Improving quality of life and reducing costs with beneficiaries
Unimed BH	MG	Health promotion centers and health care program	Self-care and monitoring of chronic patients	Improving quality of life and reducing costs with beneficiaries
Unimed-Poa	RS	*Viver bem* program	Interactive channel with information on improving quality of life for different groups, including chronic patients	Improving quality of life and reducing costs with beneficiaries
Unimed-Rio	RJ	Health promotion and disease risk prevention programs – management of chronic diseases, heart and pregnancy	Self-care and monitoring of chronic patients	Improving quality of life and reducing costs with beneficiaries
Unimed^b^ (Sta Maria, RS)	RS	Program for telemonitoring chronic patients (by phone)	Self-care and monitoring of chronic patients	Improving quality of life and reducing costs with beneficiaries

Source: Own elaboration, based on the institutional websites of the companies.

^a^ Two different strategies.

^b^ Unimed Santa Maria is not included among the largest health companies in the country; however, it was included in the universe of analysis as an example of an operator that uses telemonitoring.

Regarding the demand for the SUS, effective actions for the use of this technology are inexistent, despite FHP being a *locus* potential for the development of a broad project for remote patient monitoring, similarly to what happens with public policies. Finally, we observed that in this market, although in the embryonic phase, business agents search for new business opportunities, whereas initiatives for use by the SUS in collective health are apparently inexistent. This dichotomy can segment the provision of this service, leaving a significant part of the population at the mercy of the private payment logic.

### Challenges and Opportunities for SUS

Despite the enormous benefits of telemonitoring, few studies seek to relate its use to an improvement of the sustainability of health systems.

Based on the replicability of the most conservative data available in MEDLINE/PubMed database studies, our research resulted in an estimate of HCDST costs by SUS. Thus, Figure shows the rates of hospitalization for chronic diseases in Brazil between 2000 and 2009, which support the estimation of HCDST and respective expenses for the year 2017.

**Figure:**
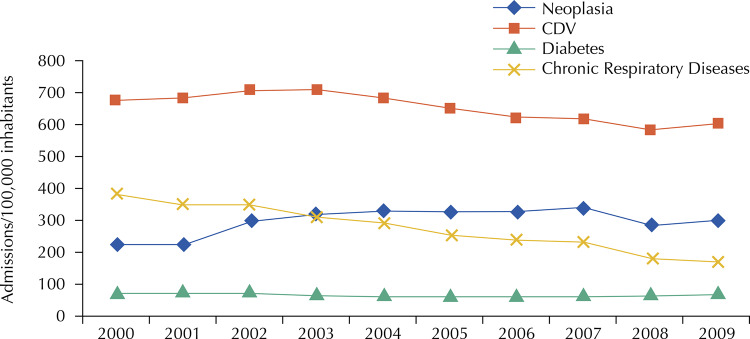
Rate of hospitalization for selected chronic diseases in Brazil, 2000 to 2009. CVD: Cardiovascular disease

Source: Prepared by the Brazilian Ministry of Health^4^ (p. 43) based on data from the SUS Hospital Information System.

### Cost Calculation for Telemonitoring Sensitive Hospitalizations in 2017

Based on Figure, we can determine the total rate of HCNCD (Tx HCNCD) per 100,000 inhabitants:

Tx HCNCD = cardiovascular diseases + cancer + diabetes + respiratory diseases = 600 + 300 + 180 + 70 = 1,150

In our study, considering that the HCDST are equal to the HCNCDT minus the hospitalizations for cancer, we can estimate the total rate of HCDST (Tx HCDST) per 100,000 inhabitants:

Tx HCDST = Tx HCNCD − Tx cancer = 1,150 − 300 = 850

With the HCDST rate, we can estimate the HCDST and its respective percentage in relation to the total number of hospitalizations (% HCDST) in 2009:

$$\mathrm{HCDST}\;=\;(\mathrm{Tx}\;\mathrm{HCDST}\;\times \;{\mathrm{population}}^{27})\;/\;100,000\;=\;(850\;\times \;190,755,799)\;/\;100,000\;=\;1,621,424$$

% HCDST in relation to total hospitalizations = 1,621,424 / 9,011,304 = 18%

In Brazil, the MH does not have historical series data available for the analysis of the SUS hospitalizations. However, the data available for the period between January 2017 and May 2017 provide important indications about the Brazilian problem^28^: the total number of hospitalizations in 2017 was 3,952,133 in May, and 9,485,119 in December, and the total cost of hospitalizations in 2017 was R$ 4,549,734,441.63 until May and R$ 10,919,362,659.91 until December.

Based on the aforementioned data and considering the percentage of HCDST (18%) estimated for 2009, expenditures on hospitalizations per HCDST can be estimated for 2017 as follows: 10,919,362,659.91 × 0.18 = R$ 1,965,485,278.78.

That is, the potential for cost reduction only with the use of telemonitoring for HCDST in 2017 is around R$ 2.0 billion if aligned with a comprehensive primary care strategy in the SUS^29^. Since our study seeks not only the potential for cost reduction, but also an estimate of the economic benefits with the adoption for HCDST by the SUS, we show some MEDLINE/PubMed study results and analyze its replicability for the Brazilian situation.

One of the pioneers in these studies is the Veteran Health Administration (VHA), a program aimed at serving about 22 million American war veterans, which developed a pilot telemonitoring program with 900 patients between 2000 and 2003. The results show a 40% reduction in emergency care, a 63% decrease in hospital admissions, an 88% reduction in home care time and a satisfaction rate of around 90% in the period^18^.

Another study conducted between 2004 and 2007 that analyzed HCNCD and hospitalizations for mental health issues pointed drastic reductions in hospitalizations due to the use of telemonitoring: 56.4% in patients with depression, 45.1% in patients suffering from posttraumatic stress disorder, 40.9% in patients suffering from other mental health problems, 30.3% in patients with hypertension, 25.9% in patients with heart failure, 20.7% in patients with chronic obstructive pulmonary disease and 20.4% in patients with diabetes. Consequently, VHA’s budget for telemedicine in 2017 increased to $1.2 billion^30^.

In two recent studies coordinated by the Maryland Health Care Commission (MHCC) involving telemonitoring, some data corroborate those found by VHA. The first analyzed the acute hospitalizations (in intensive care units) of 22 patients with CNCD at a cost of US$ 66,000.00 for the entire program. The results showed a reduction of 66% in acute hospitalizations and from 15.7% to 4.5% in the rate of readmissions. Considering the cost of US$ 10,352.00 of each hospitalization of the U.S. program *Medicare*, the estimated savings were US$ 372,672.00, much higher than the cost to implement the entire program^1^. The second lasted 180 days and only analyzed the readmissions of 57 patients with CNCD at a cost of US$ 60,000.00. The final report pointed to a reduction of 44 readmissions in the first 30 days of the program alone and an estimated savings of US$ 308,000, considering the cost of US$ 7,000 per hospitalization^2^.

Therefore, the potential for reduction was around R$ 2.0 billion in HCDST due to telemonitoring for 2017 and the variation in the rate of reduction of HCDST among the three studies above (minimum of 25% in VHA and maximum of 66% in MHCC). Hence, we estimated that costs with HCDST in the SUS could be reduced between R$ 500 million and R$ 1.3 billion per year with the replication of these experiences, without considering other benefits such as reductions in emergency room care, acute hospitalizations, readmissions and home care time, among others, which would point to an even greater economic gains.

## DISCUSSION

The MEDLINE/PubMed studies presented in our article are in consistence and point to a significant reduction in HCDST costs when compared with the costs to implement and manage telemonitoring systems. The economic benefits estimated in our research consider only the reduction of costs of HCDST for SUS. However, other important benefits can be provided by the use of telemonitoring if associated with a structure that enables a comprehensive primary care network, such as reduction in the demand for consultations and emergencies, logistics costs, costs for inactive workers, etc., as well as other qualitative benefits, such as time to respond to disorders, comfort and transportation of patients.

Retail companies are the main consumer market of specialized telemonitoring products and services, an indication that its acceptance and use by the end user grows, a result of behavioral changes in which people increasingly seek to monitor their health conditions preventively. These factors stimulate the use of telemonitoring by service providers as a powerful tool for health promotion, disease prevention, improvement of the quality of services and, especially, cost reduction. The use of this technology by large operators of private health plans, similar to global trends, will also be a major inducer for the growth of this market in the country.

Within the scope of business dynamics, solutions for the Brazilian market have been developed and offered, which demonstrates that business agents see interest and expect demand growth for these products. The interest of these business agents in this emerging market is reflected in the recent discussion on the expansion of the use of telemedicine promoted by the Federal Council of Medicine. The early launch of service packages over the private network signals both the game of interests involved and the strong pressures for greater regulatory flexibility. It also shows the tension between the private sphere, concerned with the opening of new markets, and the public sphere regarding patient safety, patient information security, doctor-patient relationship, quality or accuracy of diagnosis, among other ethical and legal aspects relevant to the practice of telemedicine. These aspects reinforce the need for a further discussion with broad participation and that contemplate the strengthening of public health in Brazil.

The country is lagging behind in telemedicine, especially in telemonitoring. Learning from other countries’ experiences, obtaining up-to-date and georeferenced data on bed use (quantity, costs and occupancy rate by type of use) and analyzing regulatory restrictions and legal aspects, safety issues and technologies most appropriate to the Brazilian reality, as well as developing contingency plans, are examples of issues that must be further studied.

Within the Scope of the SUS, we observed caution regarding new advances. Considering that the main technologies that support telemonitoring solutions also form the structural basis of other telemedicine applications, the development of a major project to reduce HCDST has the potential to progress in the construction of a comprehensive primary care network, contribute to a greater dynamism of the national productive and innovative base and induce innovations throughout the chain of this emerging industry.

Our article shows the potential of this technology, with perspectives of significant cost reduction in the SUS only considering the HCDST. However, scientific production in the specific field of telemonitoring is still insufficient. Therefore, the challenges for government agencies, service providers, industry, educational and research institutions, among others, require joining forces for the development of new studies and customized solutions to the characteristics of a country of such a continental extension; solutions oriented simultaneously to the improvement of the sustainability of the SUS, advance in the provision of health services and development of the national industry and the economic-industrial complex of health, including regarding the services already offered by Telehealth, from the perspective of the political economy of the health.
