# Supplementary material for: Healthcare telemonitoring and business dynamics: challenges and opportunities for SUS
Source: Rev Saude Publica. 2020 Jun 19;54:65. doi: 10.11606/s1518-8787.2020054001996 (PMC7304882; doi:10.11606/s1518-8787.2020054001996)
Supplement: Translation

# Translation: Telemonitoramento e a dinâmica empresarial em saúde: desafios e oportunidades para o SUS

**Authors:** Antonio da Cruz Paula, José Manuel Santos de Varge Maldonado, Carlos Augusto Grabois Gadelha

**Affiliations:** I Brasil Grupo de pesquisa cadastrado no Cnpq: apoio à gestão de tecnologia, inovações e produtos estratégicos para o Sistema de Saúde.Brasil; II Brasil Fundação Oswaldo Cruz. Escola Nacional de Saúde Pública Sérgio Arouca. Rio de Janeiro, RJ, Brasil

**Keywords:** Doenças não Transmissíveis, prevenção & controle, Telemonitoramento, Setor de Assistência à Saúde

## Abstract

**OBJETIVO:**

Apontar desafios e oportunidades para o Sistema Único de Saúde (SUS) com o uso do telemonitoramento para enfrentar o aumento crescente dos custos com as doenças crônicas não transmissíveis, a partir do seu panorama geral no Brasil, dinâmica empresarial e reaplicação de dados de estudos americanos.

**MÉTODOS:**

Este trabalho se enquadra na abordagem quali-quantitativa com pesquisa do tipo exploratória, e o trabalho de campo se concentrou na análise do mercado nacional a partir das empresas privadas, pois não se identificaram experiências ou estudos relacionados a esse tema no SUS. Para análise do panorama e da dinâmica do mercado, investigou-se a oferta a partir dos produtos e serviços disponíveis e a demanda por meio dos hospitais de referência, bem como as dez maiores empresas de planos de saúde privados. Para subsidiar a discussão central deste estudo, analisou-se a redução de custos com as internações hospitalares pelo SUS decorrentes das doenças crônicas não transmissíveis mais impactadas pelo telemonitoramento (IDCST), utilizando-se dados do Datasus e de alguns estudos americanos da base de dados MEDLINE/PubMed.

**RESULTADOS:**

Embora em fase embrionária, os agentes empresariais se posicionam na busca por novas oportunidades de negócios, enquanto na saúde coletiva não há aparentemente iniciativas públicas para o uso do telemonitoramento. A reaplicação dos dados estadunidenses implicaria uma redução significativa nos gastos com IDCST pelo SUS, sem considerar outros benefícios, tais como a redução nos atendimentos nas salas de emergência, internações agudas, reinternações e tempo de cuidados domiciliares, entre outros, que apontam para ganhos econômicos ainda superiores com o uso do telemonitoramento.

**CONCLUSÕES:**

O desenvolvimento de um grande projeto para a redução das IDCST a partir dessa tecnologia tem potencial para avançar em uma rede integral de atenção básica, contribuir para uma maior dinamização da base produtiva e inovativa nacional e induzir inovações em toda a cadeia dessa indústria emergente.

## INTRODUÇÃO

O telemonitoramento se caracteriza pelo acompanhamento remoto de dados de saúde do local onde o paciente se encontra até um centro especializado de monitoramento, interpretação e análise, sendo uma das mais importantes aplicações da telemedicina. Entre seus principais benefícios, citam-se a desospitalização, saúde preventiva, diagnóstico precoce, aumento na expectativa de vida, melhor conforto, aumento de horas trabalhadas etc. Sua utilização vem sendo considerada importante instrumento para enfrentar o crescimento dos custos com as doenças crônicas não transmissíveis (DCNT) pelos sistemas nacionais de saúde, um dos principais problemas na prestação de serviços de saúde em todo o mundo^1^.

A preocupação com as DCNT é mundial, com previsão de impactos nas metas estabelecidas nos Objetivos de Desenvolvimento do Milênio, pois são responsáveis pelas principais causas de óbitos no mundo, com reflexos na perda de qualidade de vida e limitação do trabalho e lazer, bem como nos impactos econômicos para as famílias e sistemas de saúde. Por isso, a Organização das Nações Unidas (ONU) tem orientado os países a desenvolver planos de ação para sua prevenção e controle^4,5^.

Nesse enfrentamento, a atenção básica tem um papel importante, na medida em que algumas DCNT são evitáveis por condições sensíveis à atenção primária (CSAP)^6,7^. Por isso os países buscam diferentes abordagens, dentre as quais o telemonitoramento tem se destacado como uma importante alternativa.

No Brasil, a construção do Sistema Único de Saúde (SUS) enfrenta desafios significativos, entre eles o atendimento ao preceito constitucional da sua universalização, suas dimensões continentais e sua população de mais de 200 milhões de pessoas. Não obstante a complexidade do quadro referente às transições demográfica e epidemiológica, elas implicam maiores esforços com forte impacto no orçamento da saúde. O aumento das DCNT responde por 75% dos gastos do SUS e por 72% dos óbitos, dos quais 31,3% são decorrentes de doenças do aparelho circulatório, 16,3% de câncer, 5,2% de diabetes e 5,8% de doenças respiratórias, fazendo desses quatros grupos os mais representativos^4,8^.

Em que pesem as diversas experiências e importantes estudos decorrentes do Programa Telessaúde Brasil Redes (Telessaúde), observa-se uma insuficiência na produção científica no campo específico do telemonitoramento que busca demonstrar os benefícios decorrentes do seu uso, inclusive no âmbito de custos, dada sua inegável importância para os sistemas de saúde público e privado^7,9^. Um exemplo é um estudo recente realizado no Brasil com idosos em condição crônica, que apontou uma relação positiva entre o uso do telemonitoramento e a adesão ao tratamento medicamentoso^12^.

Contudo, verifica-se o desenvolvimento e oferta de soluções para o mercado brasileiro, o que sinaliza o interesse e expectativa dos agentes empresariais no crescimento da demanda, ao mesmo tempo que se observa uma carência de políticas públicas de saúde nessa área. Tal fato aponta a necessidade de uma maior participação do Estado, sob o risco de as oportunidades abertas pelo telemonitoramento serem apropriadas exclusivamente pelas estratégias empresariais.

Dado esse quadro, o objetivo deste artigo é identificar, a partir da dinâmica empresarial e institucional e dos benefícios econômicos, os desafios e oportunidades para o SUS com o uso do telemonitoramento.

## MÉTODOS

Este trabalho se enquadra na abordagem quali-quantitativa com pesquisa do tipo exploratória. Realizou-se uma revisão da literatura que permitiu uma análise qualitativa com desdobramentos na abordagem quantitativa para estimar a redução de custos com o uso do telemonitoramento pelo SUS^13^.

No âmbito do SUS, não se identificaram experiências ou estudos relacionados a este tema na busca nas bases de dados SciELO, Capes e Google Acadêmico, bem como na análise dos projetos no período de 2009 a 2020 do Programa de Apoio ao Desenvolvimento Institucional do SUS (Proadi)^14^. Por isso, o trabalho de campo se concentrou na análise do mercado nacional a partir das empresas privadas.

Assim, em relação à oferta, realizou-se um levantamento para identificar empresas que comercializam serviços especializados e produtos de telemonitoramento no mercado brasileiro, a partir de pesquisas no buscador Google com os descritores “monitoramento remoto saúde”, “gestão da saúde”, “homecare” e “teleassistência saúde”, enquanto os fornecedores de produtos foram identificados entre as empresas expositoras na maior feira de equipamentos hospitalares do Brasil em 2017^15^.

Quanto à demanda, optou-se pelos hospitais de referência reconhecidos pelo Ministério da Saúde (MS) no triênio de 2018–2020, ou seja, as Entidades de Saúde de Reconhecida Excelência (ESRE), e selecionaram-se as dez maiores empresas de planos de saúde privados que, de modo geral, estabelecem o *modus operandi* competitivo^16,17^.

Para subsidiar a discussão central deste estudo, analisou-se a redução de custos com as internações hospitalares pelo SUS decorrentes das DCNT mais impactadas pelo telemonitoramento (cardiovasculares, diabetes e respiratórias), neste artigo denominadas internações por doenças crônicas sensíveis ao telemonitoramento (IDCST), um subgrupo das internações por DCNT (IDCNT), categoria já existente e monitorada pelo MS^1,4,18^. Para tal, utilizaram-se dados do Datasus e de alguns estudos da base de dados MEDLINE/PubMed, identificados a partir dos descritores “telemedicine”, “telehealth” e “ehealth” associados aos termos “economic benefit monitoring” e “remote patient monitoring”. Por fim, os cálculos da redução de custos com IDCST foram obtidos a partir da replicabilidade dos dados mais conservadores encontrados nesses estudos.

## RESULTADOS

Apresenta-se a seguir um quadro do telemonitoramento no Brasil com dados das principais iniciativas políticas, questões regulatórias e mercado nacional, bem como algumas estimativas de benefícios econômicos para o SUS com o uso do telemonitoramento.

### Quadro do Telemonitoramento no Brasil

Observam-se poucas ações para a ampliação da sua utilização no Brasil, especialmente pelo SUS, apesar dos imensos benefícios apontados por especialistas e da evolução da telemedicina no país nos últimos anos, fruto de incentivos das agências de fomento à pesquisa e importantes ações governamentais, que resultaram na formação de infraestrutura, equipes e núcleos de pesquisa em diversas instituições acadêmicas do país. Em relação às políticas públicas, pode-se dizer que no Brasil ocorreram algumas iniciativas que culminaram essencialmente nos projetos Rede Universitária de Telemedicina (Rute) e o Telessaúde, ambos com o foco na telemedicina para suporte e melhoramento da qualidade da Atenção Básica^9,10^.

Atualmente o Telessaúde está implantado em todos os estados do país e se constitui em importante instrumento de inovação para o Programa Saúde da Família (PSF). Não obstante a ênfase dada a ele em alguns estados, a exemplo do serviço de eletrocardiograma a distância desenvolvido em MG, telerradiografia (TeleRX) pelo RJ, telediagnóstico para doenças respiratórias crônicas e de ultrassonografias obstétricas no RS e teleducação em SP, entre outros, não se identificam ações nacionais no campo do telemonitoramento.

Entretanto, apesar de o Plano de Ações Estratégicas para o Enfrentamento das DCNT do MS prever o uso do telemonitoramento, e de estudos apontarem para seus benefícios, não se observam iniciativas no âmbito das políticas públicas de saúde no Brasil, bem como não se tem conhecimento de estudos formais do MS para uma análise de seu custo-benefício, ainda que diversos outros países, como os integrantes da União Europeia (UE), tenham essa aplicação como um dos focos principais na estratégia de combate aos problemas decorrentes desse grupo de doenças^4,19^.

Quanto aos aspectos regulatórios, há uma grande variedade no posicionamento dos conselhos federais em relação ao uso da modalidade telemedicina pelos profissionais e empresas prestadoras de serviços. Há conselhos com posicionamentos totalmente liberais e sem nenhuma regulamentação para a prestação de serviços a distância, como os de educação física e serviço social; há outros com significativa flexibilização, como os de psicologia e enfermagem; já alguns ainda são muito reativos, como os de fisioterapia, nutrição e medicina^20^. Especificamente em relação ao telemonitoramento, diferente de outras aplicações da telemedicina, no Brasil não há uma restrição regulatória explícita para o seu uso nas diversas especializações do campo da saúde.

O caso do Conselho Federal de Medicina (CFM) merece uma análise mais detalhada. Com a resolução nº 2.227 de 2018 publicada em fevereiro de 2019, o CFM ampliou as atribuições da telemedicina, viabilizando serviços médicos a distância vis-à-vis, ou seja, a teleconsulta. Ante inúmeras críticas de médicos, entidades representativas da classe e conselhos regionais, sobretudo no que diz respeito à participação insuficiente na elaboração da referida resolução, e devido ao lançamento antecipado por algumas empresas privadas de novos serviços baseados na teleconsulta, vedada até então pela resolução nº 1.643 de 2002 vigente, o CFM optou pelo adiamento de sua entrada em vigor.

Embora essa discussão tenha como elemento central a teleconsulta, ela se reflete no telemonitoramento naquilo que diz respeito à iniciativa privada, por sua capacidade de resposta, na perspectiva de ocupação desse mercado com a oferta de pacotes de serviços integrados, cujo anúncio antecipado no início de fevereiro de 2019 pelo hospital Albert Einstein ilustra este ponto. No âmbito da saúde pública, por sua vez, decisões regulatórias que não envolvam todas as partes interessadas com amplas discussões e reflexões sobre impactos das mesmas poderão desvirtuar projetos de assistência integral e fragilizar ainda mais o SUS.

Quanto ao mercado nacional, há ofertas de serviços especializados e produtos para ele, bem como uma grande variedade de dispositivos, um indicativo de que existe potencial para a comercialização dessa tecnologia no país. O Quadro 1 apresenta empresas no Brasil que oferecem serviços especializados e produtos no âmbito do telemonitoramento, a unidade federativa em que estão localizadas, aplicação e clientes^4,11^.

**Quadro 1:** Empresas fornecedoras de serviços especializados e produtos em telemonitoramento: levantamento de campo.


Websites	Fornecedores de serviços especializados				

	Empresa	UF	Produto/Serviços	Aplicação	Clientes

http://www.ocuidadorvirtual.com.br/	24/7 Care	SP	Teleassistência em geral, orientação médica à distância e chamada de ambulância	Pulseira eletrônica com botão de alarme para atendimento a idosos	Mercado varejo
https://www.athoscare.com.br/	Athoscare	SP	Prestadora de serviços de home care com telemonitoramento básico (telefone)	Diabetes, hipertensão arterial, Alzheimer, acamado ou com alguma dificuldade	Mercado varejo
http://lincare.com.br/	Lincare	MG	Monitoramento remoto via celular para idosos	Pulseira eletrônica para medição de sono, batimentos cardíacos, pressão e passos/distância percorrida	Mercado varejo
https://qualirede.com.br/	Qualirede	SC	Especializada em gestão de planos de saúde	Inteligência artificial, *data analytics, data science* e análises preditivas - gestão da saúde de 65.000 pacientes crônicos	Mercado corporativo
http://www.telehelp.com.br/	Tele Help	SP	Teleassistência em geral, orientação médica à distância e chamada de ambulância	Pulseira eletrônica com botão de alarme para atendimento a idosos	Mercado varejo
http://unicaresaude.com.br/	Unicare Saúde	SP	Prestadora de serviços no segmento de assistência domiciliar de alta complexidade	Usa a solução da i9Access	Operadoras de planos de saúde e mercado varejo
	
	**Fornecedores de produtos**				

	**Empresa**	**UF**	**Produto/Serviços**	**Aplicação**	**Clientes**
	
	Apple	Global	App Saúde e Apple Watch	Saúde preventiva com foco no incentivo e controle da alimentação, atividades físicas, sono e atividades de relaxamento	Mercado varejo - usuários de *smartphones*
	Bioaps	SP	PRM - Patiente Relantionship Management	Empoderamento do usuário e prevenção	61.000 pacientes da Seguradora de Saúde São Francisco
	Cisco/Nexa	Global	Smart Care	Conceito de plataforma de colaboração com uma central de atendimento que incentiva a adoção de hábitos saudáveis, o cumprimento de prescrições e monitora as condições de saúde dos clientes	Hospitais, clínicas e profissionais de saúde
	Fitbit	Global	Pulseiras inteligentes Fitbit	Pulseiras de monitoramento de atividades físicas	Mercado varejo
	i9Access	RS	Sistema de gestão de telemonitoramento	Telemonitoramento e empoderamento	Unicare Saúde - prestadora de serviços de home care
	Lifemed	SP	Central Lifeview com capacidade de monitorar sinais vitais até 32 pacientes	Tele-homecare, saude preventiva e DCNT	Hospitais, clínicas e profissionais de saúde
	Mcare	RJ	Sistema de telemonitoramento baseado em sistemas móveis	Obtém e gerencia dados de dispositivos via bluetooth, repassando para os profissionais de saúde por email, voz ou sms	Hospitais, clínicas e profissionais de saúde
	Philips	Global	Central de monitoramento de pacientes IntelliVue	Acesso remoto às informações de pacientes para o suporte em decisões críticas	Hospitais, clínicas e profissionais de saúde
	Signove	PB	Plataforma Sig Health, sistema de telemonitoramento integrado a diversos dispositivos	Tele-homecare, saude preventiva e DCNT	Hospitais, clínicas e profissionais de saúde
	Samsung	Global	App S Health	Saúde preventiva com foco no incentivo e controle da alimentação, atividades físicas e sono	Mercado varejo - usuários de smart fones sumsung
	Ventrix	SP	Sistemas de telemedicina	Telemonitoramento de bebês e tele-ECG	Mercado varejo e profissionais de saúde
	Xiaomi	Global	Relógios inteligentes	Relógios inteligentes para monitoramento de atividades físicas	Mercado varejo

Fonte: Elaboração própria a partir de Hospitalar^15^, Maldonado, Marques e Cruz^11^ e sites institucionais das empresas.

Constata-se diversidade em relação à modalidade de prestação de serviços especializados. Há prestadores de serviços de homecare, gerenciamento da saúde e telemonitoramento para idosos. Quanto às tecnologias utilizadas, observam-se o uso de telefone, *wearables* ou vestimentas inteligentes como pulseiras eletrônicas, bem com a aplicação de inteligência artificial e *big data* para análise preditiva e gerenciamento da saúde.

Em relação aos produtos, nota-se que há no mercado nacional a oferta de soluções alinhadas às principais estratégias apontadas pela literatura para o seu uso pelos portadores de DCNT: educação ou autocuidado, telecuidado e gerenciamento ou monitoramento. Porém o principal mercado consumidor é o varejo, um indicativo de que as prestadoras de serviço ainda não adotaram o telemonitoramento como ferramental por um lado, mas, por outro, uma sinalização de que cresce a sua aceitação pelo usuário final. Esses primeiros movimentos da iniciativa privada sinalizam perspectivas de crescimento do mercado, em que novas oportunidades de negócios, ampliação dos serviços prestados e aumento dos custos da saúde em função das DCNT, entre outros fatores, ajudam a explicar o crescente interesse nessa indústria emergente. Em que pese a divulgação de resultados de estudos em outros países, que apontam para impactos positivos do seu uso em relação à melhoria na qualidade de vida e redução de custos na prestação de serviços de saúde aos portadores de DCNT, verificam-se no Brasil algumas iniciativas das empresas privadas nessa área, focadas sobretudo no monitoramento remoto, sem o uso de tecnologias inovadoras que permitiriam o telemonitoramento remoto em tempo real.

O Quadro 2 apresenta as mais importantes prestadoras privadas de serviços de saúde do país, as quais desenvolveram iniciativas para a prevenção e gerenciamento da saúde em seus negócios, com a perspectiva de melhorar a qualidade de vida e o autocuidado dos pacientes em geral e dos pacientes crônicos em especial, ainda que com objetivos diferentes. Os hospitais apostam no desenvolvimento de um novo mercado, a medicina preventiva, para o qual construíram estruturas com equipes multidisciplinares e equipamentos, tendo como elemento central a comercialização de exames de *check-up*; já as seguradoras de saúde apresentam estruturas semelhantes, porém para a prevenção e gerenciamento da saúde, visando sobretudo a redução de custos.

**Quadro 2:** Principais iniciativas das prestadoras de serviços privados.


Hospitais de referência				

Empresa	UF	Serviço	Aplicação	Foco

Albert Einstein^a^	SP	Tele babycare, telecessação do tabagismo, Einsten em movimento e bem estar Einstein	Bebês, fumantes, atividades físicas orientadas, psicologia positiva	Autocuidado como resultado da venda de *check-up* e exames preventivos
Albert Einstein^a^	SP	Teleconsulta	Empresas, pacientes e outros prestadores de serviços de saúde	Criação de novos pacotes de serviços integrados e ampliação do mercado
Sírio Libanês	SP	Acompanhamento da saúde e check up	Medicina preventiva e doentes crônicos	Autocuidado como resultado da venda de *check-up* e exames preventivos
Hospital do Coração	SP	Programa de *check-up* para o paciente - teleconsultoria e telediagnóstico para SUS	Medicina preventiva e doentes crônicos	Autocuidado como resultado da venda de *check-up* e exames preventivos para clientes - novo negócio com serviços de telemedicina para SUS e outros hospitais
Hospital Alemão Oswaldo Cruz	SP	Modelo assistencial	Medicina preventiva	Autocuidado como resultado da venda de check up e exames preventivos

**Seguradoras e operadoras de planos provados de saúde**				
**Empresa**	**UF**	**Serviço**	**Aplicação**	**Foco**

Amil	Nacional	Gestão de paciente de alto risco e estratégia saúde da família	Monitoramento de pacientes crônicos, porta de entrada única para os pacientes com a ESF e incentivo ao autocuidado	Melhoria da qualidade de vida e redução de custo com os beneficiários
Bradesco Saúde	Nacional	Juntos pela saúde	Monitoramento de pacientes crônicos e antitabagismo	Melhoria da qualidade de vida e redução de custo com os beneficiários
Cassi	Nacional	Estratégia da família	Promoção da saúde e a prevenção de doenças	Uso racional da rede de serviços credenciados e especializados
Hapvida	Nordeste	Programas HapPrev e Viva leve	Medicina preventiva, autocuidado e monitoramento contínuo dos participantes do HapPrev - educação e monitoramento de crônicos com o Viva Leve.	Melhoria da qualidade de vida e redução de custo com os beneficiários
Intermedica	Nacional	Apoio ao paciente com doenças crônicas	Monitoramento proativo e autocuidado	Melhoria da qualidade de vida e redução de custo com os beneficiários crônicos
Nacional Unimed	Nacional	Estratégia de atenção integral da saúde e programa Gestão de saúde	Autocuidado e monitoramento de engajamento ao autocuidado para os crônicos	Melhoria da qualidade de vida em geral e redução de custo com os beneficiários
Sulamérica	Nacional	Programa Saúde ativa e aplicativo sharecare	Tecnologias inovadoras para monitorar condições de saúde e engajar beneficiários em atividades de autocuidado	Melhoria da qualidade de vida e redução de custo com os beneficiários
Unimed BH	MG	Centros de promoção da saúde e programa de atenção à saúde	Autocuidado, acompanhamento e monitoramento de crônicos	Melhoria da qualidade de vida em geral e redução de custo com os beneficiários
Unimed-Poa	RS	Programa Viver bem	Canal iterativo com informações sobre melhoria da qualidade de vida para diversos grupos, entre os quais os crônicos	Melhoria da qualidade de vida e redução de custo com os beneficiários e grupos específicos
Unimed-Rio	RJ	Programas de promoção de saúde e prevenção de riscos de doenças - gerenciamento de doenças crônicas, do coração e da gestação	Autocuidado, acompanhamento e monitoramento	Melhoria da qualidade de vida e redução de custo com os beneficiários e grupos específicos
Unimed^b^ (Sta Maria, RS)	RS	Programa de telemonitoramento de crônicos (uso do telefone)	Acompanhamento e monitoramento de crônicos	Melhoria da qualidade de vida e redução de custo com os beneficiários crônicos

Fonte: Elaboração própria a partir dos sites institucionais das empresas.

^a^ Duas estratégias diferentes.

^b^ A Unimed de Santa Maria não está inserida entre as maiores empresas de saúde do país, porém foi incluída no universo da análise como exemplo de operadora que se utiliza do telemonitoramento.

Em relação à demanda pelo SUS, semelhante ao que ocorre com as políticas públicas, observa-se até o momento a inexistência de ações efetivas para a utilização dessa tecnologia, apesar de o PSF, por exemplo, ser um *locus* em potencial para o desenvolvimento de um projeto amplo para o monitoramento remoto de pacientes crônicos. Finalizando esta análise, constata-se que neste mercado, embora em fase embrionária, os agentes empresariais se posicionam na busca por novas oportunidades de negócios, enquanto na saúde coletiva aparentemente não há iniciativas para o uso pelo SUS. Essa dicotomia pode segmentar a prestação desse serviço, deixando parte significativa da população à mercê da lógica privada de pagamento.

### Desafios e Oportunidades para o SUS

Apesar dos enormes benefícios do telemonitoramento, poucos são os estudos que buscam relacionar o seu uso à melhoria de sustentabilidade dos sistemas de saúde.

A partir da replicabilidade dos dados mais conservadores disponíveis em estudos da base de dados MEDLINE/PubMed, esta pesquisa resultou em uma estimativa de custos com IDCST pelo SUS. Assim, apresenta-se a Figura com as taxas de internação hospitalar por doenças crônicas no Brasil no período de 2000 a 2009, que subsidiam o cálculo das IDCST e respectivos gastos para o ano de 2017, descrito abaixo.

**Figura:** Taxa de internação hospitalar por doenças crônicas selecionadas no Brasil, 2000 a 2009. DCV: doença cardiovascular

Fonte: Elaborado pelo Ministério da Saúde^4^ (p. 43) a partir de dados do Sistema de Informações Hospitalares do SUS.

### Cálculo do Custo com Internações Sensíveis ao Telemonitoramento em 2017

A partir da Figura, é possível definir a taxa total de IDCNT (Tx IDCNT) por 100.000 habitantes:

Tx IDCNT = doenças cardiovasculares + câncer + diabetes + doenças respiratórias = 600 + 300 + 180 + 70 = 1.150

Considerando que neste estudo as IDCST são iguais às IDCNT menos as internações por câncer, é possível calcular a taxa total de IDCST (Tx IDCST) por 100.000 habitantes:

Tx IDCST = Tx IDCNT − Tx câncer = 1.150 − 300 = 850

Com a taxa de IDCST, é possível calcular as IDCST e seu respectivo percentual em relação ao total de internações (% IDCST) em 2009:

$$\mathrm{IDCST}\;=\;(\mathrm{Tx}\;\mathrm{IDCST}\;\times \;{\mathrm{população}}^{27})\;/\;100.000\;=\;(850\;\times \;190.755.799)\;/\;100.000\;=\;1.621.424$$

% IDCST em relação ao total de internações = 1.621.424 / 9.011.304 = 18%

No Brasil, o MS não possui dados em série histórica disponíveis para a análise do quadro de internações do SUS. Entretanto, os dados disponíveis para o período de janeiro de 2017 a maio de 2017 fornecem indicações importantes sobre o tamanho do problema brasileiro^28^: o total de internações em 2017 foi de 3.952.133 até maio ou de 9.485.119 até dezembro, e o custo total das internações em 2017 foi de R$ 4.549.734.441,63 até maio ou R$ 10.919.362.659,91 até dezembro.

A partir dos dados acima, e considerando-se mantido em 2017 o mesmo percentual das IDCST (18%) calculado para 2009, podem-se estimar os gastos com internações por IDCST para todo o ano de 2017 em: 10.919.362.659,91 × 0,18 = R$ 1.965.485.278,78.

Ou seja, o potencial de redução de custos apenas com o uso de telemonitoramento para IDCST no ano de 2017 é da ordem de R$ 2,0 bilhões, se alinhado a uma estratégia de atenção básica integral no SUS^29^. Como busca-se neste estudo não apenas o potencial de redução de custos, mas uma estimativa dos benefícios econômicos com a adoção para as IDCST pelo SUS, apresentam-se a seguir os resultados de alguns estudos da MEDLINE/PubMed e analisa-se sua replicabilidade para o caso brasileiro.

Um dos pioneiros nesses estudos é o *Veteran Health Administration* (VHA), um programa voltado para atender cerca de 22 milhões de veteranos de guerra americanos, que entre 2000 e 2003 desenvolveu um programa-piloto de telemonitoramento com 900 pacientes. Os resultados impressionam: redução de 40% no atendimento nas emergências, queda de 63% nas internações hospitalares, queda de 88% no tempo de cuidados domiciliares e taxa de satisfação em torno dos 90% no período analisado^18^.

Outro estudo entre 2004 e 2007 para analisar IDCNT e internações por questões de saúde mental apontou reduções drásticas nas hospitalizações devido ao uso do telemonitoramento: 56,4% nos pacientes com depressão, 45,1% nos que sofrem de transtorno de estresse pós-traumático, 40,9% em outros problemas de saúde mental, 30,3% em hipertensão arterial, 25,9% em insuficiência cardíaca, 20,7% em doença pulmonar obstrutiva crônica e 20,4% em diabetes. Em função desses resultados, o orçamento da VHA para a telemedicina em 2017 aumentou para US$ 1,2 bilhões^30^.

Em dois estudos recentes coordenados pela *Maryland Health Care Commission* envolvendo telemonitoramento, alguns dados corroboram os encontrados pela VHA. O primeiro analisou as internações agudas (em unidades de tratamento intensivo) de 22 pacientes com DCNT a um custo de US$ 66.000,00 para todo o programa. Os resultados apontaram para uma redução de 66% nas internações agudas e redução na taxa de reinternações de 15,7% para 4,5%. Considerando o custo de cada internação do programa americano *Medicare*, de US$ 10.352,00, a economia estimada foi de US$ 372.672,00, valor muito superior ao despendido para o custeio de todo o programa^1^. O segundo teve duração de 180 dias e analisou exclusivamente as reinternações de 57 pacientes com DCNT a um custo de US$ 60.000,00. O relatório final apontou para uma redução de 44 reinternações apenas nos primeiros 30 dias do programa e uma economia estimada de US$ 308.000,00, considerando-se US$ 7.000,00 por internação^2^.

Portanto, considerando-se o potencial de redução de IDCST com o telemonitoramento em torno de R$ 2,0 bilhões para 2017 e a variação na taxa de redução de IDCST entre os três estudos acima (mínimo de 25% no VHA e máximo de 66% no MHCC), pode-se estimar que, com a replicação dessas experiências, seria possível uma redução nos gastos com as IDCST no SUS entre R$ 500,0 milhões e R$ 1,3 bilhões ao ano, isso sem considerar outros benefícios, tais como reduções nos atendimentos nas salas de emergência, nas internações agudas, nas reinternações e no tempo de cuidados domiciliares, entre outras, que apontam para ganhos econômicos ainda superiores com o seu uso.

## DISCUSSÃO

Os estudos da MEDLINE/PubMed apresentados neste artigo são convergentes e apontam para uma redução significativa dos gastos com IDCST em relação aos custos necessários para implantar e gerenciar sistemas de telemonitoramento. Os benefícios econômicos estimados neste estudo levam em consideração apenas a redução de gastos com as IDCST no SUS. Entretanto, se associado a uma estruturação que viabilize uma rede de atenção básica integral, outros importantes benefícios podem ser proporcionados pelo uso do telemonitoramento, tais como redução na demanda por consultas e emergências, nos custos com logística, nos custos com trabalhadores inativos etc., bem como outros de caráter qualitativo, como tempo de resposta a distúrbios, conforto e transporte dos pacientes.

O principal mercado consumidor dos produtos e serviços especializados de telemonitoramento é o varejo, um indicativo de que cresce a sua aceitação e uso pelo usuário final, fruto inclusive de mudanças comportamentais em que as pessoas crescentemente buscam monitorar suas condições de saúde de forma preventiva. Isso tende a estimular a utilização pelos prestadores de serviços, como uma poderosa ferramenta para a promoção da saúde, prevenção de agravos, melhoria da qualidade dos serviços e sobretudo redução de custos. Acredita-se que o uso desta tecnologia pelas grandes operadoras de planos privados de saúde, à semelhança das tendências mundiais, será também um grande indutor para o crescimento desse mercado no país.

No âmbito da dinâmica empresarial, verifica-se o desenvolvimento e oferta de soluções para o mercado brasileiro, o que demonstra que os agentes empresariais apostam que há interesse e expectativa de crescimento de demanda para esses produtos. O interesse desses agentes empresariais neste mercado emergente se reflete na recente discussão sobre a ampliação do uso da telemedicina no âmbito do CFM. O lançamento antecipado de pacotes de serviços pela rede privada sinaliza tanto o jogo de interesses envolvidos como as fortes pressões para uma maior flexibilização da regulação. Mostra igualmente a tensão entre a esfera privada, preocupada com a abertura de novos mercados, e a esfera pública naquilo que diz respeito à segurança do paciente, segurança da informação do paciente, relação médico-paciente, qualidade ou acurácia do diagnóstico, entre outros aspectos éticos e legais pertinentes à prática da telemedicina. Tais aspectos reforçam a necessidade de novas reflexões com ampla participação e que contemplem o fortalecimento da saúde pública no Brasil.

O país está atrasado em relação aos benefícios da telemedicina em geral e do telemonitoramento em particular. Aprender com experiências de outros países, obter dados atualizados e georreferenciados no uso dos leitos (quantidade, custos e taxa de ocupação por tipo de utilização) e analisar as restrições regulatórias e aspectos legais, as questões de segurança e as tecnologias mais apropriadas para a realidade brasileira, bem como desenvolver planos de contingência, são exemplos de questões que necessitam ser discutidas e aprofundadas em novos estudos.

No âmbito do SUS, observa-se uma falta de ousadia para novos avanços. Na medida em que as principais tecnologias que suportam as soluções de telemonitoramento também formam a base estrutural de outras aplicações da telemedicina, o desenvolvimento de um grande projeto para a redução das IDCST tem potencial para progredir na construção de uma rede integral de atenção básica, contribuir para uma maior dinamização da base produtiva e inovativa nacional e induzir inovações em toda a cadeia dessa indústria emergente.

Este artigo aponta para o potencial dessa tecnologia, com perspectivas de redução expressiva de custos no SUS apenas com as IDCST, ao mesmo tempo em que se observa uma insuficiência na produção científica no campo específico do telemonitoramento. Portanto, os desafios que se colocam para os órgãos de governo, prestadores de serviços, indústria, instituições de ensino e pesquisa, entre outros, requer unir esforços para o desenvolvimento de novos estudos e soluções customizadas às características deste país de extensão continental que sejam orientados simultaneamente para a melhoria da sustentabilidade do SUS, avanço na prestação dos serviços de saúde e desenvolvimento da indústria nacional e do complexo econômico-industrial da saúde, inclusive em relação aos serviços já ofertados pelo Telessaúde, na perspectiva da economia política da saúde.
